# It is worth 10 million working hours a year to have your toilet paper folded?

**DOI:** 10.1186/s12995-016-0126-5

**Published:** 2016-12-12

**Authors:** Rickard Ljung, Hedvig Ljung, Harald Ljung

**Affiliations:** 1 Unit of Epidemiology, Institute of Environmental Medicine, Karolinska Institutet, Nobels Väg 13, SE-171 77 Stockholm, Sweden; 2 Stocksundsskolan, SE-182 73 Stocksund, Sweden

**Keywords:** Cleaning staff, Socioeconomic position, Occupational health, Psychosocial health, Inequalities, Toilet paper, Working conditions

## Abstract

**Background:**

From our experience the toilet paper is folded in the bathrooms in rooms in branded hotels. We aimed to study the total time yearly spent in the world on folding hotel toilet paper.

**Method:**

Three investigators clocked 60 folding toilet paper events and calculated the mean time. The mean folding time was 5.73 s (interquartile range 4.50–6.56). Using the calculated extra time it takes to fold the toilet paper and the number of hotel nights spent we estimated the total time spent in the world each year to fold the toilet paper. For sensitivity analyses we used different assumptions on number of hotel beds, occupancy rate and folding time.

**Results:**

Assuming an extra 10 s spent on folding toilet paper, approximately 10 million hours are globally spent on folding toilet paper every year. This corresponds to more than 5000 man-years of work. In a hotel with yearly full coverage of 200 beds skipping folding the toilet paper corresponds to around 200 h of time that could be spent elsewhere.

**Conclusion:**

To take away unnecessary duties from hotel room cleaners would increase their health and well-being and save time that could be better spent. Is it really defendable and appropriate that someone else has spent time on folding the toilet paper you are just about to use?

## Background

From our experience the toilet paper is folded in the bathrooms in rooms in branded hotels. By folding the toilet paper the hotel cleaner documents that the bathroom has been cleaned and gives an impression of that no one else has used the toilet. We aimed to study the total time yearly spent in the world on folding hotel toilet paper.

## Material, Methods and Results

There are approximately 15 million beds in branded hotels in the world [1] The daily occupancy rate has varied between 50–70 % [2]. It is difficult to exactly quantify how many hotel bathrooms are cleaned daily where the toilet papers also is folded. However, a rough estimate yields at least 10 million cleaned branded hotel rooms every day [1, 2]. In an attempt to quantify the time it takes to fold toilet paper three investigators (RL, HeL, HaL) clocked 60 folding events and calculated the mean time. The mean folding time was 5.73 s (interquartile range 4.50–6.56). The clocking started with the folder facing away from the toilet paper holder and ended in the same position.

Using the calculated extra time it takes to fold the toilet paper and the number of hotel nights spent we estimated the total time spent in the world each year to fold the toilet paper. For sensitivity analyses we used different assumptions on number of hotel beds, occupancy rate and folding time (Table 1).

**Table 1:** Total time spent annually in the world on folding toilet paper in bathrooms in branded hotels. Estimation based on different assumptions on number of hotel beds, occupancy rate and folding time

Estimated number of branded beds	Estimated average daily occupancy rate	Days cleaned per year	Folding time	Total time spent on folding toilet paper
N	%	Days	Sec	Hours
15 million	50	365	5	3.8 million
15 million	50	365	10	7.6 million
15 million	70	365	5	5.3 million
15 million	70	365	10	10.6 million
20 million	50	365	5	5.1 million
20 million	50	365	10	10 million
20 million	70	365	5	7.1 million
20 million	70	365	10	14.2 million

## Discussion

Assuming an extra 10 s spent on folding toilet paper, approximately 10 million hours are globally spent on folding toilet paper every year. This corresponds to more than 5000 man-years of work. In a hotel with yearly full coverage of 200 beds skipping folding the toilet paper corresponds to around 200 h of time that could be spent elsewhere.

The study has some limitations; the calculations were based on an approximate number of hotel beds with bathroom cleaned daily and where the toilet paper is also folded. Also, the clocked folding exercise was done by three investigators not trained as cleaning staff. However, our hands were dry and we did not have gloves and clocked just the folding time. Hence, we anticipate the actual extra time to be more than our average of 5.73 s. Our calculations are solely based on hotel rooms; we have no information on how many of the world’s non-hotel toilet facilities have a folded toilet paper after being cleaned. Also, it would be interesting to know how often people fold the toilet paper at home before having guests for dinner.

## Conclusion

Hotel room cleaners have a physically demanding work load. Even within this manual worker group there are inequalities in occupational health [3]. Also psychosocial work factors influences the physical health of hotel room cleaners [4]. To take away unnecessary duties would increase the health and well-being of the cleaning staff and save time that could be better spent. Is it really defendable and appropriate that someone else has spent time on folding the toilet paper you are just about to use?
